# Rebamipide for Managing Dyspeptic Symptoms During Proton-Pump Inhibitor Washout Before *Helicobacter pylori* Testing: A Randomized, Double-Blind, Placebo-Controlled Trial

**DOI:** 10.5152/tjg.2025.25248

**Published:** 2025-10-12

**Authors:** Nottawan Suksai, Rachaneeporn Chueansuwan, Somchai Yongsiri, Raweewan Witoon, Anothai Juttuporn

**Affiliations:** 1 Division of Gastroenterology, Department of Medicine, Burapha University Faculty of Medicine, Chonburi, Thailand; 2 Division of Nephrology, Department of Medicine, Burapha University Faculty of Medicine, Chonburi, Thailand; 3 Department of Preventive Medicine and Family Medicine, Burapha University Faculty of Medicine, Chonburi, Thailand

**Keywords:** Dyspepsia, *Helicobacter pylori*, Rebamipide

## Abstract

**Background/Aims:**

Persistent dyspeptic symptoms are common during the proton pump inhibitor (PPI) washout period before *Helicobacter pylori* (*H*.* pylori*) testing. However, the role of rebamipide in symptom management during this interval remains unclear.

**Materials and Methods:**

This double-blind, randomized controlled trial enrolled 65 patients with *H*.* pylori*–associated dyspepsia or gastritis, randomized (1:1) to receive rebamipide (100 mg 3 times daily) or placebo for 4 weeks, following a 14-day eradication regimen. The primary outcome was the proportion of responders achieving a ≥25% reduction in pain symptom scores on the Severity of Dyspepsia Assessment scale at week 6. Secondary outcomes included changes in pain symptoms, non-pain symptoms, and dyspepsia-related health scores, as well as eradication rates and safety.

**Results:**

All patients completed the trial. Although the proportion of responders was higher in the rebamipide group (18 patients, 56.3% vs. 13 patients, 39.4%), this difference was not statistically significant (*P* = .17). Scores for pain, non-pain, and dyspepsia-related health improved similarly in both groups. Eradication rates were comparable (87.5% vs. 90.0%), and no serious adverse events were reported.

**Conclusion:**

The responder rate was higher in the rebamipide group, but the difference did not reach statistical significance. The potential benefit of rebamipide as a rescue therapy during the PPI washout period before *H*.* pylori* testing warrants further investigation in larger trials.

**Main Points**

- Many patients continue to experience dyspeptic symptoms during the proton pump inhibitor washout period, when most symptom-relieving medications must be withheld before *Helicobacter pylori* (*H*. *pylori*) testing.
- The responder rate was higher in the rebamipide group, but the difference did not reach statistical significance.
- Rebamipide did not interfere with *H*.* pylori* eradication and was well tolerated throughout the study.

## Introduction

The global prevalence of *Helicobacter pylori (H*.* pylori)* infection remains high, making it a leading cause of chronic gastritis and dyspepsia, which are both common clinical conditions.^1^^,^^2^ The diagnostic gold standard is esophagogastroduodenoscopy (EGD) with gastric mucosal biopsy for histopathological staining. Other reliable diagnostic methods include the rapid urease test, urea breath test (UBT), and stool antigen test.^3^^-^^5^

Treatment of *H*.* pylori*–induced gastritis typically involves a combination of antibiotics and proton pump inhibitors (PPIs), supported by several evidence-based regimens.^3^^,^^6^^-^^8^ Post-treatment eradication is essential and is usually assessed using noninvasive tests. During the required waiting period of at least 4 weeks before testing with the UBT, stool antigen test, or urease test, all antibiotics and most symptom-relieving medications, including PPIs, vonoprazan, antacids, alginates, and bismuth-containing compounds, must be avoided due to their potential to cause false-negative results.^3^^,^^9^^,^^10^ However, many patients continue to experience a considerable burden of dyspeptic symptoms during this period.^3^^,^^11^

Rebamipide is a mucoprotective agent that stimulates prostaglandin synthesis and promotes mucus glycoprotein production, helping protect the gastric epithelium.^12^ In *H*.* pylori* infection, it has shown benefits including reduced proinflammatory cytokine production, histological improvement with long-term use, symptom relief in *H*.* pylori*–associated ulcers, and increased eradication rates when combined with proton pump inhibitor (PPI) and antibiotic dual therapy.^12^^-^^15^ Importantly, rebamipide does not interfere with diagnostic tests such as the UBT, stool antigen test, or urease test.^10^ Given these properties, it was hypothesized that rebamipide may serve as a rescue therapy for symptom control during the PPI washout period. However, no randomized trials have specifically evaluated this approach. This study aimed to assess the efficacy of rebamipide in managing dyspeptic symptoms during the PPI washout phase preceding eradication testing.

## Materials and Methods

### Study Design

**Figure 1.**
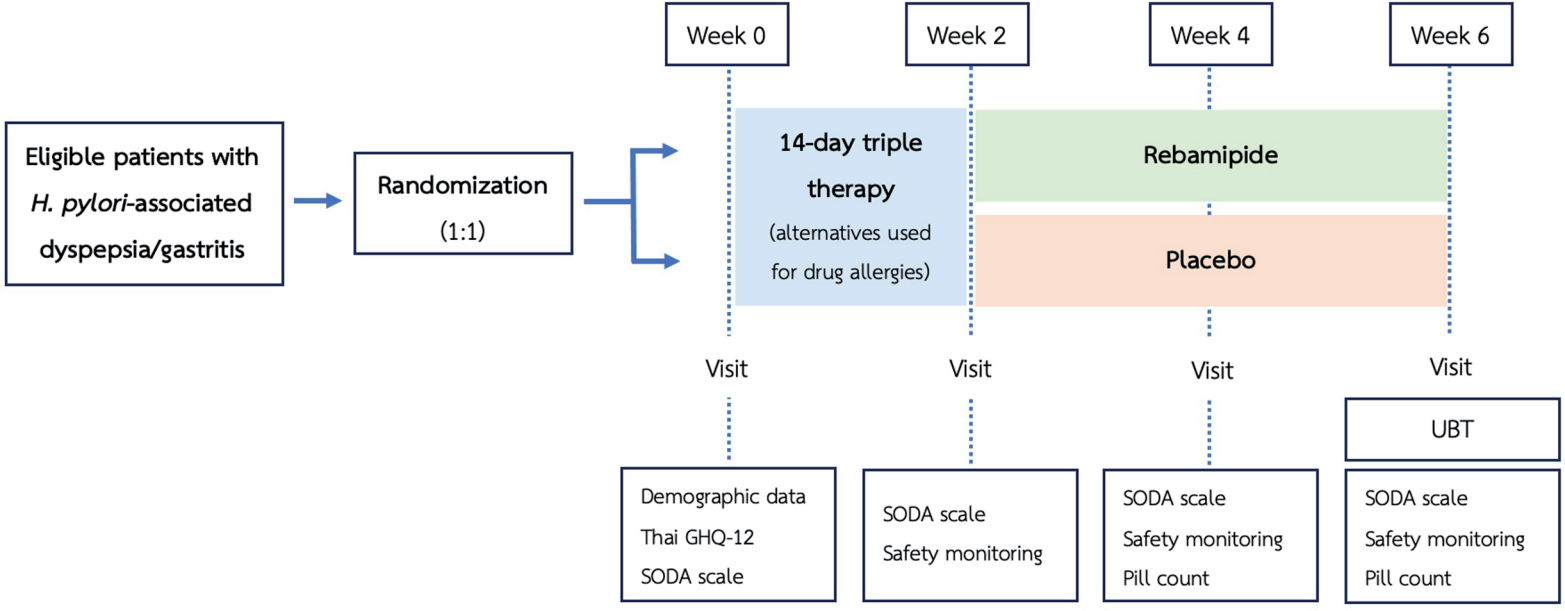
Flowchart of the study interventions and assessments. *H*. *pylori*, *Helicobacter pylori*; Thai GHQ-12, Thai General Health Questionnaire-12; SODA scale, Severity of Dyspepsia Assessment scale; UBT, urea breath test.

This was a prospective, single-center, double-blind, randomized controlled trial (RCT) conducted between September 5, 2024, and February 28, 2025. The study protocol received ethics committee approval from the Burapha University Ethics Committee on August 15, 2024, in accordance with the Declaration of Helsinki (approval No: IRB1-082/2567). Written informed consent was obtained from all participants prior to data collection. A flowchart of the study interventions and assessments is shown in Figure 1.

### Participants

The study enrolled Thai patients aged 18 years or older who were diagnosed with *H*.* pylori*–associated dyspepsia or gastritis through a UBT, stool antigen test, or endoscopic evaluation using a urease test or histological staining. Esophagogastroduodenoscopy was performed based on clinical indications determined by the attending physician prior to enrollment. Exclusion criteria included a history of allergy to rebamipide, use of rebamipide within 1 month before enrollment, documented active peptic ulcers that had not healed, uncontrolled psychiatric disorders, symptomatic gallstones, unresolved cancers, ongoing substance abuse, and pregnancy or breastfeeding. Withdrawal criteria were defined as severe allergic reactions to rebamipide, inability to attend scheduled follow-up visits, or voluntary withdrawal from the study.

### Randomization and Interventions

Eligible patients were randomly assigned to 1 of 2 groups using block randomization with a block size of 4 (1:1 ratio). The rebamipide group received rebamipide 100 mg 3 times daily after meals (Thai Otsuka Pharmaceutical Co., Ltd.), while the placebo group received a matching placebo. Both rebamipide and placebo tablets were identical in appearance and were dispensed in identical, light- and moisture-protective packaging to ensure blinding. Allocation was concealed from both investigators and participants.

During the first 2 weeks, all patients in both groups were treated with a 14-day triple therapy regimen for *H*.* pylori* eradication. The regimen consisted of amoxicillin 1000 mg twice daily after meals, clarithromycin 500 mg twice daily after meals, and omeprazole 20 mg twice daily before meals. For patients allergic to amoxicillin, metronidazole was used as a substitute. Those with an allergy to omeprazole were given vonoprazan as an alternative. In cases of clarithromycin allergy, a 14-day levofloxacin-based therapy was used instead of the clarithromycin-based regimen.

After completing the *H*.* pylori* eradication regimen, patients in the rebamipide group continued taking rebamipide for an additional 4 weeks, while those in the placebo group received a placebo for the same duration. At the end of this period, all patients underwent a UBT (POConePlus®, Otsuka Pharmaceutical Co., Ltd., Tokyo, Japan) to assess *H*.* pylori* eradication status.

Participants received detailed instructions on treatment benefits, risks, and procedures. Throughout the study, participants were instructed to avoid over-the-counter medications, except for prokinetic agents, which were permitted as rescue therapy. The use of any PPI was prohibited during the study period except during the first 2 weeks of eradication therapy. In addition, vonoprazan, antacids, alginates, and bismuth-containing compounds were not allowed due to their potential to affect the accuracy of *H*.* pylori* testing. All concomitant medications for underlying conditions were reviewed for potential drug interactions with study medications, and any potentially harmful combinations were avoided. Adherence to the regimen was evaluated by pill counts at weeks 4 and 6.

### Outcomes and Data Collection

The primary outcome was the efficacy of rebamipide versus placebo in managing dyspeptic symptoms during the PPI washout phase before *H*.* pylori* eradication testing. This was evaluated by comparing the proportion of responders between groups. Responders were defined as patients achieving a ≥25% reduction in pain symptom scores on the Severity of Dyspepsia Assessment (SODA) scale^16^ from baseline to the end of treatment.

The secondary outcomes were to evaluate the efficacy of rebamipide in improving individual symptom domains and overall dyspepsia-related health, as measured by the 3 components of the SODA scale: pain symptoms, non-pain gastrointestinal symptoms, and dyspepsia-related health scores. Additional secondary outcomes included comparisons of *H*.* pylori* eradication rates between groups and assessments of the drug’s safety profile.

Patient data were collected on Days 0, 14, 28, and 42. On Day 0, baseline characteristics (including age, sex, weight, height, body mass index, comorbidities, history of nonsteroidal anti-inflammatory drugs (NSAIDs) or antiplatelet use, and lifestyle factors such as smoking, alcohol consumption, irregular eating habits, and excessive caffeine intake) were recorded. A detailed dyspepsia history, including symptom duration and prior medications, was also documented. The method of *H*.* pylori* diagnosis was noted, and for patients who had undergone EGD, both endoscopic and histological findings were recorded. The severity of dyspeptic symptoms, including both pain and non-pain components as well as dyspepsia-related health, was assessed using the SODA scale. Mental health was evaluated using the Thai version of the General Health Questionnaire-12 (GHQ-12) to screen for psychological disorders.^17^^,^^18^ On Days 14, 28, and 42, the SODA scale was reassessed, and adverse medication reactions were recorded. A 24-hour contact number was also provided for patients to report any adverse events throughout the study.

The SODA scale is a well-validated tool widely used for assessing dyspepsia.^16^^,^^19^^,^^20^ It consists of 17 items across 3 domains: pain symptoms (score range: 2-47), which assess abdominal pain severity; non-pain symptoms (score range: 7-35), which evaluate upper gastrointestinal issues such as belching, heartburn, bloating, excessive flatulence, bitter taste, nausea, and halitosis; and the dyspepsia-related health score (score range: 2-23), which reflects the impact of symptoms on well-being. Lower scores in the pain and non-pain domains indicate less severe symptoms, whereas higher dyspepsia-related health scores indicate better well-being. Mental health status was assessed using the Thai GHQ-12, a validated screening tool for psychiatric disorders in the Thai population. It comprises 12 items, with a score of 2 or higher indicating a potential psychiatric disorder.

### Statistical Analysis

This pilot RCT evaluated the preliminary efficacy of rebamipide versus placebo during the PPI washout period. Based on feasibility considerations and pilot trial guidelines,^21^ the aim was to enroll approximately 30 participants per group (total n = 60), without performing a formal sample size calculation. According to Julious,^21^ a minimum of 12 subjects per group is generally sufficient to estimate means and variances with acceptable precision in early-phase studies. Analyses were primarily based on the intention-to-treat (ITT) principle, including all randomized participants. A per-protocol (PP) analysis was also conducted, including only participants who completed the study and demonstrated ≥80% adherence.

Patient characteristics were summarized using means, medians, or frequencies. Between-group comparisons of responder proportions were assessed using the chi-square or Fisher’s exact test. Continuous variables were analyzed using the independent *t*-test or Mann–Whitney *U* test. Changes in SODA scale scores were analyzed using generalized estimating equations (GEEs). A two-sided *P*-value < .05 was considered statistically significant.

## Results

### Patient Characteristics

Sixty-five patients with *H*.* pylori*–associated dyspepsia or gastritis were randomized into 2 groups (rebamipide: n = 32; placebo: n = 33). Demographic and clinical characteristics are presented in Table 1, and additional baseline variables such as comorbidities, lifestyle-related risk factors, and diagnostic methods are shown in Supplementary Table 1. Baseline SODA scale scores are shown in Table 2. The mean age was 51 years, with 27 (41.5%) males and 38 (58.5%) females. The mean duration of dyspeptic symptoms was 3 months. The use of dyspepsia-relieving medications, NSAIDs, or antiplatelets, as well as the presence of lifestyle-related risk factors for dyspepsia, was comparable between groups. Mental health screening (Thai GHQ-12) identified 7 participants in each group with abnormal scores, with no notable group difference. Baseline characteristics were generally balanced, except that the rebamipide group had higher baseline pain scores and lower dyspepsia-related health scores.

Most patients were diagnosed with *H*.* pylori* infection via EGD with a urease test. A history of EGD with histological evaluation was documented in 81.4% of the rebamipide group and 84.8% of the placebo group (*P* = .70). Endoscopic findings included erosive or hemorrhagic gastritis in 46.9% of the rebamipide group and 60.6% of the placebo group (*P* = .27), and non-erosive gastritis in 21.9% and 12.1%, respectively (*P* = .29). Histological examination revealed moderate-to-severe chronic active gastritis in 71.9% vs. 75.8% (*P* = .72), and complete or incomplete intestinal metaplasia in 3.1% vs. 12.1% (*P* = .20). Endoscopic and histological profiles did not differ significantly between groups.

### Treatment Regimen and Adherence

All patients received 14-day triple therapy, except for 1 in the rebamipide group who was treated with a levofloxacin-based regimen (Table 3). Study drug adherence exceeded 80% in all participants. Full adherence was achieved in 28 patients in the rebamipide group and 30 in the placebo group. Among those who missed doses, the maximum number of missed days was 5.

As all randomized participants completed the study and maintained ≥80% adherence to the assigned treatment, the PP population was identical to the ITT population. Accordingly, the efficacy results from both analyses were consistent.

### Helicobacter pylori Eradication and Symptom Changes (Severity of Dyspepsia Assessment Scale)

All patients underwent UBT 4 weeks after completing *H*.* pylori* treatment, followed by the administration of rebamipide or placebo. The overall *H*.* pylori* eradication rate was not significantly different between groups, with rates of 87.5% in the rebamipide group and 90.9% in the placebo group (*P* = .66; Table 3).

**Figure 2.**
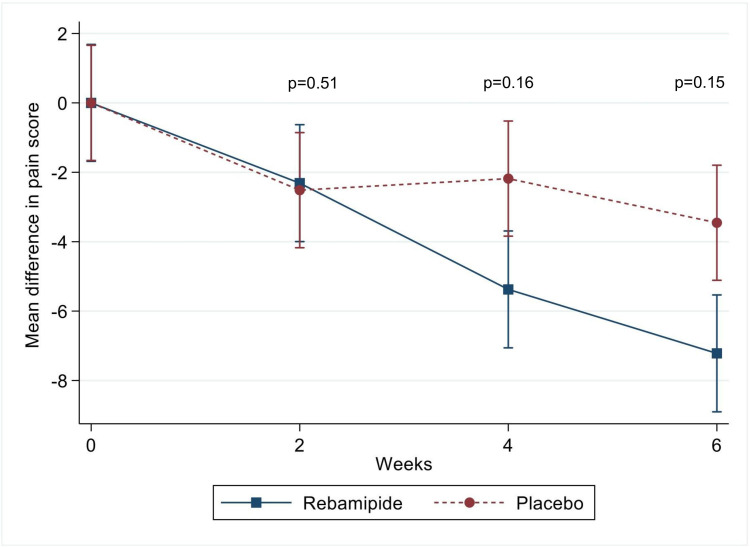
Mean change in pain symptom scores in the rebamipide and placebo groups. Line graph shows changes in pain scores from baseline at weeks 2, 4, and 6, measured using the Severity of Dyspepsia Assessment scale. The rebamipide group had a greater pain reduction than the placebo at weeks 4 and 6, but the differences were not significant. Group comparisons used generalized estimating equations, adjusted for baseline with ANCOVA. Analyses were conducted on the intention-to-treat population. Error bars indicate SDs.

Changes in SODA scale scores over time and between-group comparisons are summarized in Table 4. The first SODA scale component, which measures pain symptoms (score range: 2 to 47; lower scores indicate less pain), was assessed at baseline and weeks 2, 4, and 6. Mean scores in the rebamipide group were 24.44 ± 4.35, 22.13 ± 6.03, 19.06 ± 6.09, and 17.22 ± 4.67, respectively. In the placebo group, scores were 20.82 ± 8.56, 18.3 ± 7.97, 18.64 ± 8.9, and 17.36 ± 8.96 at corresponding time points. Pain scores significantly decreased in both groups. In unadjusted comparisons, the rebamipide group showed greater reductions at weeks 4 and 6, with mean differences of 3.19 (95% confidence interval [CI]: 0.60-5.79; *P* = .02) and 3.76 (95% CI: 1.17-6.36; *P* = .004), respectively. However, after adjusting for baseline differences using ANCOVA, these between-group differences were no longer statistically significant (adjusted *P* = .16 and .15 at weeks 4 and 6, respectively), as shown in Figure 2.

**Figure 3.**
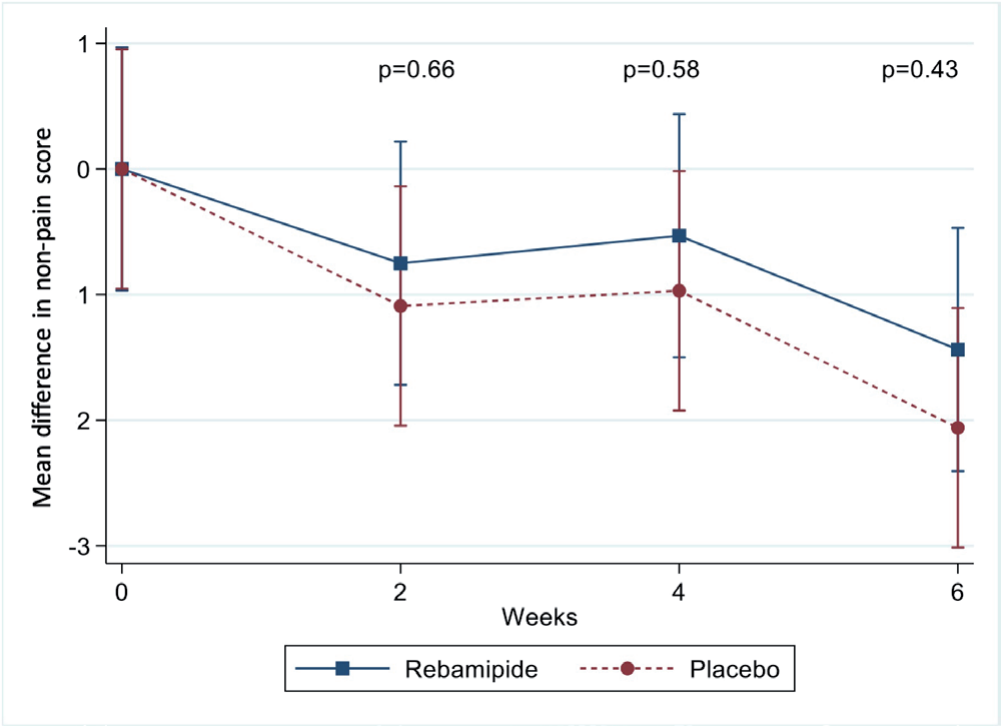
Mean change in non-pain symptom scores in the rebamipide and placebo groups. The line graph illustrates changes in non-pain symptom scores from baseline at weeks 2, 4, and 6, based on the Severity of Dyspepsia Assessment scale. Although both groups showed symptom improvement, the between-group differences were not statistically significant at any time point. Group comparisons were analyzed using generalized estimating equations. Statistical analysis was conducted on the intention-to-treat population. Error bars indicate SDs.

The second component of the SODA scale, non-pain symptoms, decreased in both groups, indicating overall improvement. Scores declined from 15.88 ± 3.46 to 14.44 ± 2.72 in the rebamipide group (*P* = .005), and from 15.52 ± 2.9 to 13.45 ± 3.28 in the placebo group (*P* < .001). However, the between-group difference in score reduction was not statistically significant (Figure 3).

**Figure 4.**
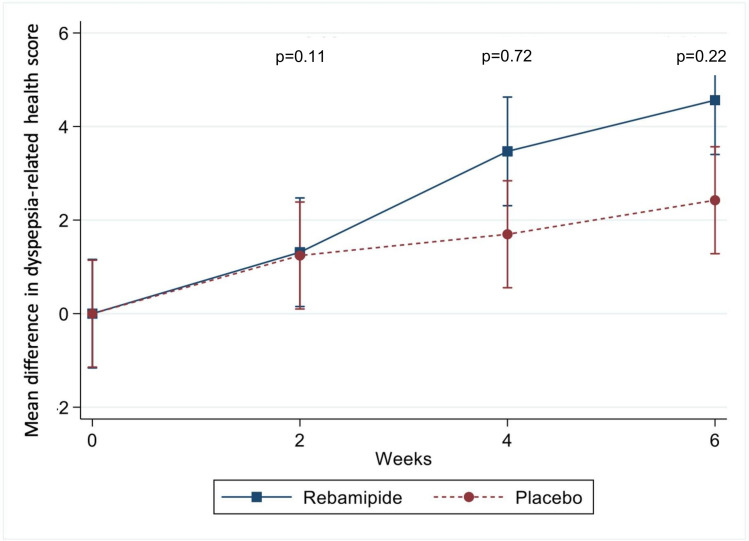
Mean change in dyspepsia-related health scores in the rebamipide and placebo groups. Line graph showing mean changes in dyspepsia-related health scores from baseline at weeks 2, 4, and 6, measured using the Severity of Dyspepsia Assessment scale. Scores improved over time, but between-group differences were not significant after adjustment. Group comparisons used generalized estimating equations, adjusted for baseline with ANCOVA. Analyses were conducted on the intention-to-treat population. Error bars indicate SDs.

The third SODA component, dyspepsia-related health (score range: 2-23), improved in both groups over 6 weeks. Mean scores increased from 9.5 to 14.06 in the rebamipide group and from 11.27 to 13.70 in the placebo group. Unadjusted comparisons showed greater improvement in the rebamipide group at weeks 4 and 6 (*P* = .04 and .01, respectively). Nonetheless, statistical significance was lost after baseline adjustment (adjusted *P* = .72 and .22; Figure 4).

### Dyspepsia Response Rate

The dyspepsia response rate, defined as the proportion of patients with at least a 25% improvement in the pain symptom score of the SODA scale from baseline to week 6, was higher in the rebamipide group (18 patients, 56.3%) than in the placebo group (13 patients, 39.4%). However, the difference was not statistically significant (*P* = .17).

### Safety Data

No serious adverse events occurred, as detailed in Table 3. During the initial 2 weeks of *H*.* pylori* treatment, adverse effects such as metallic taste, nausea, vomiting, and dizziness were comparable between groups. From weeks 2 to 6, when patients received rebamipide or placebo, no adverse symptoms were reported in the rebamipide group. In contrast, the placebo group reported 1 case of each of the following symptoms: nausea, constipation, headache, and palpitations.

## Discussion

This double-blind, RCT evaluated the efficacy of rebamipide in reducing dyspeptic symptoms during the mandatory washout period for PPIs, vonoprazan, alginates, bismuth compounds, and antibiotics before *H*.* pylori* testing. The study found no significant difference between groups in the proportion of patients achieving ≥25% improvement in pain symptoms, and no significant differences across the 3 SODA scale domains (pain, non-pain, and dyspepsia-related health).

The absence of a statistically significant benefit for the primary outcome may be due to several factors. First, the effects of rebamipide on reducing mucosal inflammation may require a longer treatment period to yield clinical improvements. Second, rebamipide may not directly target acid or gas-related mechanisms that could provide more rapid symptom relief. Third, it may be less effective for addressing visceral hypersensitivity and gut–brain interactions, which are often implicated in patients with functional dyspepsia. As a result, the role of rebamipide as a short-term rescue therapy may be limited.

Of note, in the rebamipide group, the unadjusted results showed greater reductions in pain scores at weeks 4 and 6, along with improvements in dyspepsia-related health. Although these differences were no longer significant after adjustment, the findings of this pilot trial may still provide meaningful insights into the therapeutic potential of rebamipide. Larger, adequately powered studies are required to confirm these observations. By contrast, no trend of improvement was observed for non-pain symptoms (e.g., bloating, belching, and nausea). These findings raise the possibility that any short-term benefit of rebamipide may be more specific to pain-dominant dyspepsia. However, this remains speculative and warrants further investigation.

The hypothesis that rebamipide may benefit pain-dominant dyspepsia is based on its pharmacological properties, including the stimulation of prostaglandin production, enhancement of mucosal protection, reduction of oxidative stress, and promotion of epithelial regeneration.^12^ These effects may be more effective in targeting visceral pain and mucosal sensitivity than in alleviating motility or gas-related symptoms. If future studies confirm these benefits, this specificity may help guide individualized dyspepsia treatments based on patient-reported symptom profiles.

Comparable eradication rates between groups suggest that the observed outcomes were related to the efficacy of the study treatment, rather than differences in microbial clearance. Notably, abdominal pain persisted in both groups after 6 weeks despite high eradication rates. This finding is consistent with prior observations that dyspeptic symptoms often continue despite successful eradication therapy,^3^^,^^11^ potentially due to residual mucosal inflammation or coexisting functional dyspepsia. Functional dyspepsia is a complex disorder involving delayed gastric emptying, impaired accommodation, visceral hypersensitivity, and altered central processing of gut signals, often influenced by psychological comorbidities.^22^ These pathophysiological factors may underlie persistent symptoms after eradication, highlighting the need for targeted, symptom-based treatments during and beyond the diagnostic washout period.

While rebamipide has been studied as an adjunct in *H*.* pylori* eradication regimens, especially in patients with peptic ulcer, earlier studies have demonstrated its benefits in enhancing ulcer healing.^23^ Long-term use has also been associated with histological improvements in gastritis and reductions in serum gastrin.^24^ In terms of eradication efficacy, rebamipide has been shown to improve *H*.* pylori* eradication rates only when used in dual therapy, but not in triple therapy or other currently recommended regimens,^25^ a finding consistent with the results of this study. At present, rebamipide has not been granted an approved indication for *H*.* pylori* eradication by international consensus.^3^^,^^6^^-^^8^ However, evidence regarding its role in symptomatic control remains limited. This study aimed to address this gap by evaluating the clinical effects of rebamipide on patient-reported dyspeptic symptoms using standardized eradication regimens and validated symptom assessment tools.

Strengths of this study include its double-blind RCT design, high treatment adherence, use of validated outcome measures, and 100% follow-up. Moreover, the pilot design enabled the generation of preliminary effect size estimates to inform future larger-scale trials. Limitations include the relatively small sample size, single-center design, short follow-up duration, and lack of long-term outcome data. In addition, pathological assessment was incomplete in some participants. Future studies should recruit larger samples, evaluate whether benefits persist beyond the washout period, and assess efficacy in other patient subgroups, such as those with post-eradication functional dyspepsia or more severe baseline inflammation.

Rebamipide did not show a significant advantage over placebo in alleviating dyspeptic symptoms during the PPI washout period prior to *H*.* pylori* testing.

## Supplementary Materials

Supplementary Material

## Figures and Tables

**Table 1.** Baseline Characteristics of the Study Population

Demographic Characteristics	Rebamipide Group (n = 32)	Placebo Group (n = 33)	*P*
Age (years)^†^	50.32 ± 16.97	51.14 ± 16.11	.84
Sex (male), n (%)^‡^	13 (40.6)	14 (42.4)	.88
Body mass index (kg/m^2^)^†^	24.94 ± 5.75	24.44 ± 4.65	.70
Duration of dyspeptic symptoms (months)*	3.5 (2, 10)	3 (1, 5)	.17
NSAID or antiplatelet use, n (%)^‡^	6 (18.8)	5 (15.2)	.70
EGD performed, n (%)^‡^	26 (81.3)	28 (84.8)	.70
Endoscopic finding			
Erosive/hemorrhagic gastritis, n (%)^‡^	15 (46.9)	20 (60.6)	.27
Non-erosive gastritis, n (%)^‡^	7 (21.9)	4 (12.1)	.29
Other, n (%)^‡^	8 (25)	9 (27.3)	.84
Pathological findings			
Moderately/severe chronic active gastritis, n (%)^‡^	23 (71.9)	25 (75.8)	.72
Mildly/non-active gastritis, n (%)^‡^	3 (9.4)	2 (6.1)	.62
Intestinal metaplasia (complete/incomplete), n (%)^‡^	1 (3.1)	4 (12.1)	.20
Potential psychiatric disorders assessed by Thai GHQ-12, n (%)^‡^	7 (21.9)	7 (21.2)	.95

*P*-values were calculated using the independent t-test or the Mann–Whitney *U* test for continuous variables, and the chi-square or Fisher’s exact test for categorical variables. Statistical significance was defined as *P* < .05. Analysis was conducted based on the intention-to-treat population.

Additional baseline characteristics are presented in Supplementary Table 1.

EGD, esophagogastroduodenoscopy; NSAIDs, nonsteroidal anti-inflammatory drugs; Thai GHQ-12, Thai General Health Questionnaire-12.

^†^Data are displayed as the mean ± SD.

*Median (interquartile range).

^‡^Number (%).

**Table 2.** Severity of Dyspepsia Assessment Scale at Baseline

SODA Scale	Rebamipide Group (n = 32)	Placebo Group (n = 33)	*P*
Pain symptom score	24.44 ± 4.35	20.82 ± 8.56	.04
Non-pain symptom score	15.88 ± 3.46	15.52 ± 2.9	.65
Dyspepsia-related health score	9.5 ± 2.42	11.27 ± 2.49	.005

Data are displayed as the mean ± SD. *P*-values were calculated using the independent *t*-test. Statistical significance was defined as *P* < .05. Analysis was conducted based on the intention-to-treat population.

SODA scale, Severity of Dyspepsia Assessment scale.

**Table 3.** *Helicobacter pylori* Treatment Regimens, Eradication Outcomes, and Adverse Drug Reactions

Variable	Rebamipide Group (n = 32)	Placebo Group (n = 33)	*P*
*H*.* pylori* treatment regimens			
14-day triple therapy, n (%)	31 (96.9)	33 (100)	.31
14-day levofloxacin-based regimen, n (%)	1 (3.1)	0 (0)	.31
*H*.* pylori* eradication rate (negative UBT, n%)	28 (87.5)	30 (90.9)	.66
Prokinetic use during trial, n (%)	4 (12.5)	2 (6.1)	.37
Adverse drug reactions of *H*.* pylori* treatment regimen, n (%)	4 (12.5)	8 (24.2)	.22
Metallic taste, n (%)	2 (6.25)	4 (12.12)	
Nausea or vomiting, n (%)	1 (3.13)	2 (6.06)	
Dizziness, n (%)	1 (3.13)	1 (3.03)	
Diarrhea, n (%)	0 (0)	1 (3.03)	
Adverse drug reactions of study drug (rebamipide or placebo, n (%))	0 (0)	4 (12.1)	.053
Nausea, n (%)	0 (0)	1 (3.03)	
Constipation, n (%)	0 (0)	1 (3.03)	
Headache, n (%)	0 (0)	1 (3.03)	
Palpitation, n (%)	0 (0)	1 (3.03)	

Data are displayed as the number (%).

*P*-values were calculated using the chi-square test or Fisher’s exact test, as appropriate. Statistical significance was defined as *P* < .05. Analysis was conducted based on the intention-to-treat population.

*H*. *pylori*, *Helicobacter pylori*; UBT, urea breath test.

**Table 4.** Changes in Severity of Dyspepsia Assessment Scale Scores Over Time and Between-Group Differences in the Rebamipide and Placebo Groups

SODA Scale	Rebamipide Group (n = 32)			Placebo Group (n = 33)			Difference Between Groups		
	Mean ± SD	Mean Change (95% CI)	*P*	Mean ± SD	Mean Change (95% CI)	*P*	Mean Difference (95% CI)	*P*	Adjusted Mean Difference^†^
**Pain Score**									
Baseline	24.44 ± 4.35	Reference	1	20.82 ± 8.56	Reference	1	–	–	–
Week 2	22.13 ± 6.03	−2.31 (−4, −0.63)	.007*	18.3 ± 7.97	−2.52 (−4.48, −0.55)	.01*	−0.2 (−2.8, 2.39)	.88	0.51
Week 4	19.06 ± 6.09	−5.38 (−7.06, −3.69)	<.001*	18.64 ± 8.9	−2.18 (−4.15, −0.21)	.03*	3.19 (0.6, 5.79)	.02*	0.16
Week 6	17.22 ± 4.67	−7.22 (−8.9, −5.54)	<.001*	17.36 ± 8.96	−3.45 (−5.42, −1.49)	.001*	3.76 (1.17, 6.36)	.004*	0.15
**Non-Pain Score**									
Baseline	15.88 ± 3.46	Reference	1	15.52 ± 2.9	Reference	1	–	–	–
Week 2	15.13 ± 3.56	−0.75 (−1.76, 0.26)	.15	14.42 ± 3.53	−1.09 (−2.24, 0.06)	.06	−0.34 (−1.88, 1.2)	.66	–
Week 4	15.34 ± 2.84	−0.53 (−1.54, 0.48)	.30	14.55 ± 4.23	−0.97 (−2.12, 0.18)	.10	−0.44 (−1.98, 1.1)	.58	–
Week 6	14.44 ± 2.72	−1.44 (−2.45, −0.42)	.005*	13.45 ± 3.28	−2.06 (−3.21, −0.91)	<.001*	−0.62 (−2.16, 0.91)	.43	–
**Dyspepsia-Related Health Score**									
Baseline	9.5 ± 2.42	Reference	1	11.27 ± 2.49	Reference	1	–	–	–
Week 2	10.81 ± 2.83	1.31 (0.3, 2.32)	.01*	12.52 ± 3.62	1.24 (−0.04, 2.53)	.06	−0.07 (−1.71, 1.57)	.93	0.11
Week 4	12.97 ± 3.64	3.47 (2.46, 4.48)	<.001*	12.97 ± 3.58	1.7 (0.41, 2.98)	.01*	−1.77 (−3.41, −0.13)	.04*	0.72
Week 6	14.06 ± 3.61	4.56 (3.55, 5.57)	<.001*	13.7 ± 4.01	2.42 (1.14, 3.71)	<.001*	−2.14 (−3.78, −0.5)	.01*	0.22

Data are presented as mean ± SD and mean change with 95% confidence intervals (CI). Mean differences were calculated as the score in the rebamipide group minus that in the placebo group. *P*-values were calculated using the Generalized Estimating Equation.

^†^Adjusted for baseline score using ANCOVA. *Statistically significant at *P* < .05.

SODA scale, Severity of Dyspepsia Assessment Scale.

## Data Availability

The datasets generated and/or analyzed during the current study are not publicly available due to hospital and ethics committee regulations but are available from the corresponding author upon reasonable request.
