# Supplementary material for: Rebamipide for Managing Dyspeptic Symptoms During Proton-Pump Inhibitor Washout Before Helicobacter pylori Testing: A Randomized, Double-Blind, Placebo-Controlled Trial
Source: Turk J Gastroenterol. 2025 Oct 12;37(2):251–9. doi: 10.5152/tjg.2025.25248 (PMC12910302; doi:10.5152/tjg.2025.25248)
Supplement: Supplementary Material [file supplementary_material.pdf]

Supplementary Table 1. Additional baseline characteristics of the study population

Demographic characteristics	Rebamipide group (n=32)	Placebo group (n=33)	p-value
Comorbidities[§]			
Diabetes mellitus, *n* (%)[‡]	16 (50%)	19 (57.6%)	0.54
Hypertension, *n* (%)[‡]	11 (34.4%)	14 (42.4%)	0.51
Gastro-esophageal reflux disease, *n* (%)[‡]	8 (25%)	7 (21.2%)	0.72
Constipation, *n* (%)[‡]	4 (12.5%)	3 (9.1%)	0.66
Other, *n* (%)[‡]	11 (34.4%)	14 (42.4%)	0.505
Previous PPI use, n (%)[‡]	21 (65.6%)	18 (54.5%)	0.36
High-volume alcohol consumption, n (%)[‡]	0 (0%)	0 (0%)	NA
Excessive caffeine intake, n (%)[‡]	18 (56.3%)	22 (66.7%)	0.39
Irregular eating habits, n (%)[‡]	23 (71.9%)	22 (66.7%)	0.65
H. pylori diagnostic method			
Histological staining, n (%)[‡]	12 (37.5%)	9 (27.3%)	0.38
Urease test, n (%)[‡]	14 (43.8%)	19 (57.6%)	0.27
Urea breath test, n (%)[‡]	6 (18.8%)	4 (12.1%)	0.46
Stool *H. pylori* antigen, n (%)[‡]	0 (0%)	1 (3%)	0.32

Data are displayed as the mean ± standard deviation (SD)[†] and number (%).[‡]
p-values were calculated using the independent t-test or the Mann–Whitney U test for continuous variables, and the chi-square or Fisher's exact test for categorical variables. Statistical significance was defined as $p < 0.05$. Analysis was conducted based on the intention-to-treat population.
[§]These data were obtained from medical records and documented diagnoses.
PPI, proton-pump inhibitor; *H. pylori*, *Helicobacter pylori*
